# Dual role of SND1 facilitates efficient communication between abiotic stress signalling and normal growth in *Arabidopsis*

**DOI:** 10.1038/s41598-018-28413-x

**Published:** 2018-07-04

**Authors:** Chan Young Jeong, Won Je Lee, Hai An Truong, Cao Sơn Trịnh, Joo Yeon Jin, Sulhee Kim, Kwang Yeon Hwang, Chon-Sik Kang, Joon-Kwan Moon, Suk-Whan Hong, Hojoung Lee

**Affiliations:** 1 Department of Biosystems and Biotechnology, College of Life Sciences and Biotechnology, Korea University, Anam-dong 5-ga, Seongbuk-gu, Seoul 136-713 Republic of Korea; 2 Institute of Life Science and Natural Resources, Korea University, Seoul, 136-713 Republic of Korea; 3 Crop Breeding Division, National Institute of Crop Science, RDA, 181 Hyeoksin-ro, Iseo-myeon, Wanju-gun, Jeollabuk-do 54955 Republic of Korea; 4 Department of Plant Life and Environmental Sciences, Hankyong National University, 327 Jungangro, Anseong, 17579 Republic of Korea; 5 Department of Molecular Biotechnology, College of Agriculture and Life Sciences, Bioenergy Research Center, Chonnam National University, Gwangju, Republic of Korea

## Abstract

Certain plant cells synthesize secondary cell walls besides primary cell walls. This biosynthesis is strictly controlled by an array of transcription factors. Here, we show that SND1, a regulator of cell-wall biosynthesis, regulates abscisic acid (ABA) biosynthesis to ensure optimal plant growth. In *Arabidopsis*, the lack of SND1 and its homolog NST1 leads to the deficiency of secondary cell walls, preventing *snd1nst1* double mutant seedlings from growing upright. Compared to wild type seedlings, the *snd1* knockout mutant seedlings accumulated less anthocyanin and exhibited low tolerance to salt stress. Compared to wild type seedlings, the *snd1* knockout seedlings were more sensitive to salt stress. Although SND1 can bind to the promoter of *Myb46*, we observed that SND1 binds directly to the promoter of the *ABI4* gene, thereby reducing ABA levels under normal growth conditions. Thus, plants adjust secondary cell wall thickening and growth via SND1. SND1 has a dual function: it activates the Myb46 pathway, fostering lignin biosynthesis to produce sufficient cell wall components for growth, while maintaining a low ABA concentration, as it inhibits growth. This dual function of SND1 may help plants modulate their growth efficiently.

## Introduction

In addition to primary cell walls, plant cells also have secondary walls, composed of cellulose, lignin, and other molecules^1^. Because only certain types of plant cells can deposit secondary cell wall materials, including phenylpropanoid, during specific developmental phases, phenylpropanoid biosynthesis is strictly controlled by an array of genes^2–5^, which have been targeted to modify lignin content in order to manipulate biomass composition, as well as plant tolerance to abiotic stress^6–8^. Diverse transcription factors (TF) modulate various compounds in the phenylpropanoid biosynthesis pathway. AtMyb46 and its homologs AtMyb83, AtMyb58 and AtMyb63, play crucial roles in cell-wall biosynthesis^9^. Furthermore, NAC (NAM, ATAF1/2, and CUC2)-domain TFs are also xylem-associated, and 105 NAC genes with numerous functions exist in the genome of *Arabidopsis thaliana*^10^. Among these, the secondary wall-associated NAC domain protein 1 (SND1) is expressed in fibre-associated cells and plays a central role in fibre thickening^3^.

Complex and multifaceted signalling cascades, accompanied by various cellular responses, are activated in plants to ensure their survival under conditions of abiotic stress. Plant cells produce osmoprotectants to compensate for the water loss caused by salt, drought, or cold stress^11^. High salinity and drought may cause a marked imbalance in redox homeostasis, which drives plant cells to reorganize primary as well as secondary metabolism^12,13^. In this process, plant hormones serve as crucial integrators that modulate complex developmental and stress signalling pathways. Among them, abscisic acid (ABA) enables *Arabidopsis* to adapt to detrimental conditions imposed by abiotic stress and often triggers the inhibition of plant growth, thereby re-directing nutrients for successful withstanding of the specific stress conditions^14^.

Anthocyanins are recognized as part of the defence mechanism that plants use when challenged by stress. Indeed, they often accumulate in response to stress^7^. We aimed to verify whether SND1, the master controller of cell-wall biosynthesis, has any role under plant stress, as expression of *SND1* is known to affect the accumulation of lignins which are produced from the same precursor of anthocyanins. Herein we report that SND1 directly regulates ABA biosynthesis to procure best possible plant growth under salinity stress. Furthermore, we show that SND1 binds directly to the promoter of the *ABI4* gene, leading to low levels of ABA under saline conditions. Our observations suggest that plants can adjust secondary cell-wall thickening and growth performance via this SND1 regulatory effect, which displays a dual function by thickening secondary walls, while concomitantly reducing ABA content when environmental conditions are favourable plant growth.

## Results

### Altered anthocyanin content in the *snd1ko* mutant and *SND1*-overexpressing line and *SND1* was induced by abiotic stresses

In our previous study, we showed that several genes involved in flavonoid biosynthesis participate in plant abiotic stress tolerance^7,8^. Plants accumulate a wide variety of flavonoids via phenylalanine through elaborate regulatory mechanisms^15^. There are several junctions in this pathway, leading to the synthesis of different types of flavonoid compounds. For example, coumaroyl CoA, which is utilized to produce anthocyanins via various enzymes including chalcone synthase (CHS)^16^, can be converted into lignins by hydroxycinnamoyl transferase (HCT). Thus, the synthesis of anthocyanin likely affects the synthesis of lignin, which belongs to the flavonoid family. SND1 is essential for the synthesis of lignin, and thereby for the formation of secondary cell walls^3^.

To determine whether the changes in lignin accumulation due to the changes in SND1 expression affect anthocyanin synthesis, we obtained seeds of the *snd1ko* mutant from TAIR and examined abiotic stress tolerance of this line. We then measured the anthocyanin content in the *snd1ko* mutant and in the *SND1*-overexpressing (*SND1-OE*) (Fig. S1). Notably, the anthocyanin content in four-day-old seedlings was lower in the mutant and higher in the *SND1*-overexpressing line than in the Col-0 wild type (WT). Under normal conditions, SND1 serves as a positive regulator of lignin synthesis. Therefore, overexpression of *SND1* should theoretically increase lignin accumulation and decrease anthocyanin accumulation. In contrast, lignin content is expected to decrease and anthocyanin content to increase in the mutant. A similar observation has been previously reported, whereby the overexpression of *SND1* reduced lignin biosynthesis^3^. These results indicate that SND1 is positively involved in the accumulation of anthocyanin. The expression of most flavonoid-related genes increased in the *SND1*-overexpressing line, but decreased in the *snd1nst1* double mutant (Fig. S2). NST1 is a homologue of SND1^1,17^, and marked effects on secondary wall biosynthesis are observed when both are deleted. Furthermore, we observed a decrease in the expression of *PAP1* in the *snd1nst1* double mutant (Fig. S2), which specifically activates the expression of genes associated with flavonoid synthesis. These results show that SND1 plays a positive role in the expression of genes associated with flavonoid biosynthesis, thereby increasing anthocyanin accumulation.

**Figure 1:**
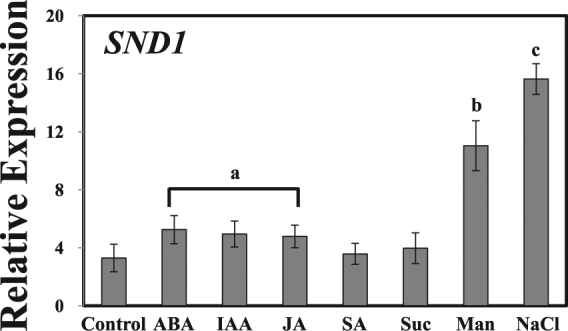
Relative expression of the *SND1* transcript in plants subjected to different hormone treatments or abiotic stresses. The relative expression of *SND1* transcript in Col-0 was determined by the qRT-PCR. Eight-day-old seedlings were used to extract mRNA following the treatment with 10 µM abscisic acid (ABA), 10 µM indole-3-acetic acid (IAA), 10 µM jasmonic acid (JA), 10 µM salicylic acid (SA), 300 mM sucrose (Suc), 400 mM mannitol (Man), and 300 mM NaCl for 6 h. The error bars indicate the standard error (SE) of three replicates. The values with different letters were significantly different from that of WT plants (P < 0.05).

Anthocyanin is a part of the plant defence mechanism. In fact, anthocyanin often accumulates in response to stress^7^. We verified whether SND1 has a role in plant stress responses, as the expression of *SND1* is known to affect the accumulation of anthocyanin. Thus, the transcript level of *SND1* was measured in Col-0 by the qRT-PCR, upon treatment with different plant hormones and stresses. In the presence of salt or mannitol, the expression of *SND1* increased. Similar results were observed with abscisic acid (ABA) treatment (Fig. 1). In particular, the expression of *SND1* was significantly higher under salinity stress than under other stress conditions tested. These results indicate that SND1 is related to osmotic stress, especially in response to salinity stress, as well as to secondary cell wall synthesis.

### Reduced tolerance of the *snd1ko* mutant under salinity stress

**Figure 2:**
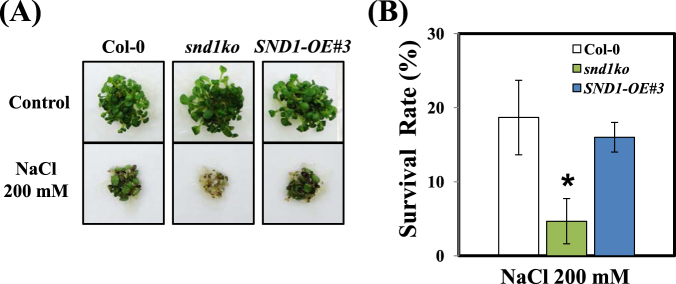
Phenotype and survival rates of Col-0, *snd1ko* mutant, and *SND1*-overexpressing line under salinity stress. (**A**) The phenotype of Col-0, *snd1ko* mutant, and *SND1*-overexpressing line under salinity stress. The seeds were sown on filter paper placed on normal medium. After 2 d, the filter paper was transferred on medium supplemented with 200 mM NaCl. The figure shows seedlings 20 d after the transfer. (**B**) Survival was quantified by counting the green cotyledons of seedlings. The experiments included 40 seeds per sample and the error bars indicate the standard error (SE) from three replicates. The asterisks represent significant differences with respect to Col-0 (P < 0.05).

As an extension of the above experiments, we verified whether altered anthocyanin biosynthesis due to *SND1* mutation can affect abiotic stress responses in plants. As shown in Fig. 2A and B, the *snd1ko* mutant exhibited a very low survival rate under salinity stress, whereas that of the *SND1*-overexpressing line did not significantly differ from the WT. The WT showed a 20% survival rate 7 d after being transferred to the 200 mM NaCl condition, whereas the mutant showed a maximal survival rate of 5% under similar conditions. However, the difference between the *SND1*-overexpressing line and WT was negligible. By the seedling-transfer method, the phenotypes were confirmed under salinity stress. The *snd1ko* mutant exhibited a lower survival rate than the WT, whereas the survival rate of *SND1*-complementation (*SND1-Com*) line was a similar to that of the WT (Fig. S3). These results indicate that the changes in *SND1* expression affect the tolerance to salinity stress in plants.

**Figure 3:**
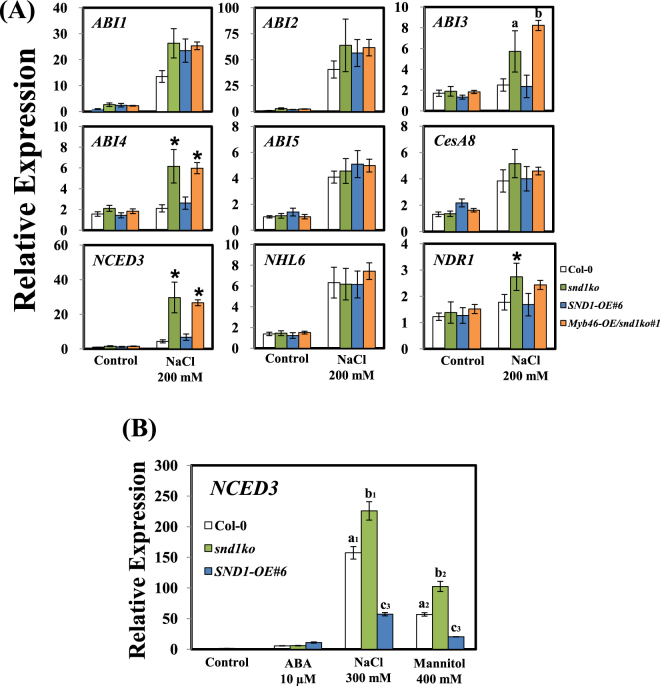
Transcript level of ABA-related genes in Col-0, *snd1ko* mutant, and transgenic plants in response to osmotic stress. (**A**) By the qRT-PCR, the relative expression of *ABI1*, *ABI2*, *ABI3*, *ABI4*, *ABI5*, *CesA8*, *NCED3*, *NHL6*, and *NDR1* was determined in Col-0, *snd1ko* mutant, *SND1*-overexpressing line, and *Myb46-*overexpressing line/*snd1ko* after treatment with 200 mM NaCl for 6 h. (**B**) The relative expression of *NCED3* in Col-0, *snd1ko* mutant, and *SND1*-overexpressing line under 10 µM ABA, 300 mM NaCl, or 400 mM Mannitol for 6 h. Eight-day-old seedlings were sampled for the analysis. The error bars indicate the standard error (SE) of three replicates. The asterisks represent significant differences from that of Col-0. The values with different letters were significantly different from that of WT plants (P < 0.05).

To determine whether the altered sensitivity to salt stress in the *snd1ko* mutant resulted from the changes in the expression of ABA-responsive genes, the expression of these genes was determined by qRT-PCR in eight-day-old seedlings. In the *snd1ko* mutant, the expression of most ABA-responsive genes increased, particularly that of *NCED3*^18^, which encodes the major enzyme involved in ABA synthesis, and was induced under salinity stress (Fig. 3A). In the experiments conducted with mannitol and NaCl, the *snd1ko* mutant exhibited higher expression of *NCED3*, whereas the expression was lower in the *SND1*-overexpression line than in the WT (Fig. 3B). These results suggest that the expression of ABA-responsive genes is negatively regulated by SND1, which might affect the biosynthesis of ABA due to the changes in *NCED3* expression.

As SND1 is known to bind directly to the *Myb46* and *Myb83* promoters to enhance lignin biosynthesis^4^, we verified whether the enhanced transcript levels of ABA-responsive genes were due to these Myb genes in the *snd1ko* mutant. Thus, we generated a transgenic plant overexpressing *Myb46* in the background of the *snd1ko* mutant, resulting in *Myb46-OE*/*snd1ko* seedlings. As the expression of ABA-related genes in *Myb46-OE*/*snd1ko* seedlings was similar to that of the *snd1ko* mutant, we concluded that Myb46 is not associated with the function of SND1, at least in terms of salt stress response (Fig. 3A).

### Enhanced ABA accumulation in the *snd1ko* mutant than the wild type

**Figure 4:**
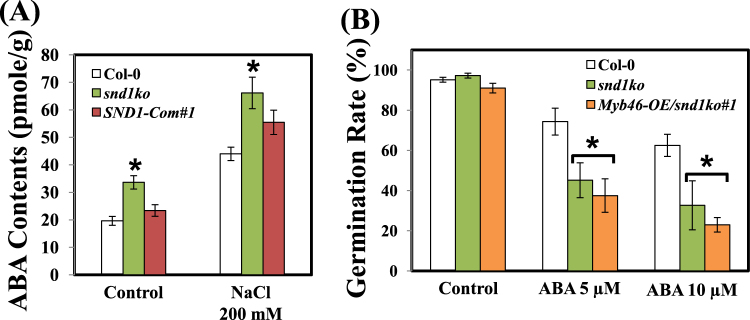
Abscisic acid (ABA) contents and germination rate of Col-0, *snd1ko* mutant, and transgenic plants. (**A**) The ABA content in Col-0, *snd1ko* mutant, and *SND1*-complementation line. Eight-day-old seedlings were used to extract ABA following treatment with 200 mM NaCl for 24 h. (**B**) Germination rate of Col-0, *snd1ko* mutant, and *Myb46-*overexpressing line/*snd1ko* in response to 5 or 10 µM ABA. The appearance of roots was regarded as germination 8 d after seeding on media supplemented with 5 or 10 µM ABA. The experiments included 120 seeds per sample, and the error bars indicate the standard error (SE) of three replicates. The asterisks represent significant differences from that of Col-0 (P < 0.05).

As NCED3 belongs to the ABA biosynthesis pathway, we determined ABA content both in eight-day-old seedlings of the *snd1ko* mutant and *SND1*-complimentation (*SND1-Com*) line following treatment with NaCl. As observed for gene expression, the amount of ABA in the *snd1ko* mutant increased under both normal and salinity conditions; however, there was no significant difference between the *SND1*-complimentation and the WT lines (Fig. 4A). These results indicate that SND1 is involved in ABA signalling, as well as in the expression of ABA-responsive genes and in the regulation of ABA accumulation.

As *SND1* regulates ABA accumulation, we verified whether the *snd1ko* mutant exhibits altered germination rates in response to ABA. To test this, we germinated Col-0, *snd1ko*, and *Myb46-OE*/*snd1ko* mutants in the presence of different concentrations of ABA in the medium (Fig. 4B). The *snd1ko* mutant showed lower germination rate in response to ABA than that in the WT. We could not revert this rate to that of WT even with the overexpression of *Myb46* in the *snd1ko* mutant background. This indicates that SND1 is associated with ABA sensitivity during germination, as well as stress tolerance. Moreover, Myb46 is not associated with the function of SND1 in ABA response.

### Direct binding of SND1 to the *ABI4* promoter

**Figure 5:**
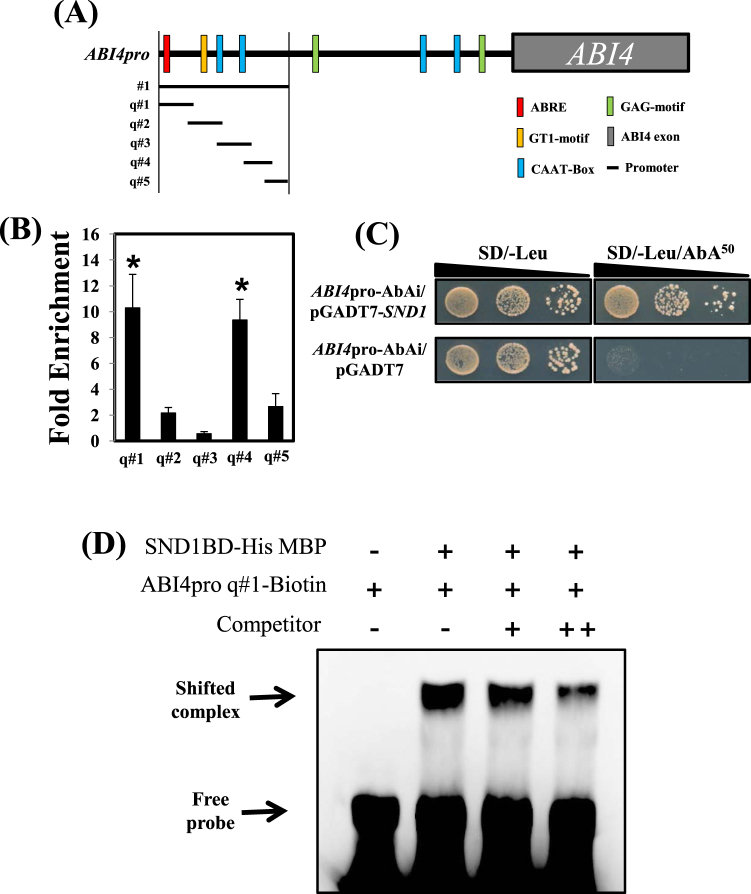
Structure of the *ABI4* promoter and chromatin immunoprecipitation (ChIP) using a SND1-GFP fusion protein. (**A**) *cis*-elements in the *ABI4* promoter region. ABRE is an abscisic acid (ABA)-response element; GT1-motif and GAG-motif are light-response elements; CAAT-Box is a common *cis*-acting element. The *PlantCARE* program was used for *ABI4* promoter analysis (http://bioinformatics.psb.ugent.be/webtools/plantcare/html/)^19^. ABI4pro#1, #1–1, and #1–2 show the size and location of the expected PCR products from the DNA template of ChIP. ABI4pro q#1, q#2, q#3, q#4, and q#5 present the size and location of the expected products of qRT-PCR products from the DNA template of ChIP. (**B**) The fold-enrichment values were calculated by dividing the ChIP signals obtained via the qRT-PCR by the signals obtained for the mock. The error bars indicate the standard error (SE). The asterisks represent significant differences among values (P < 0.05). (**C**) The yeast one-hybrid assay between SND1 and ABI4 promoter. Full-length SND1 was fused with GAL4 AD, and ABI4pro#1 was used as a bait. The ABI4pro-AbAi/pGADT7 yeast served as the negative control. Yeasts were cultured on SD/-Leu or SD/-Leu with 50 ng/mL aureobasidin A (AbA). (**D**) The electrophoretic mobility shift assay (EMSA) showed that the SND1 NAC domain (SND1 BD-His MBP) can bind to the biotin-labelled *ABI4* promoter q#1.

As the *snd1ko* mutant accumulated more ABA than that of the WT, we reasoned that SND1 may control the expression of genes associated with ABA biosynthesis. This led us to examine whether SND1 binds to the promoter of *NCED3* as its transcript level was enhanced in the *snd1ko* mutant (Fig. 5). We then performed chromatin immunoprecipitation (ChIP) to determine if SND1 can bind directly to the promoter of the *NCED3* gene. Contrary to our expectation, SND1 did not bind to the *NCED3* promoter (data not shown). We further tested the promoters of other genes (ABA anabolism-related genes: *ZEP*, *SDR1*, and *AAO3*; and a catabolism-related gene: *CYP707A3*) by the ChIP assay, but found no binding activity. However, when ABA signalling-related genes, including *ABI3* and *ABI4*, were subjected to the ChIP assay, SND1 clearly bound to the promoter of the *ABI4* gene, but not to the promoter of *ABI3* (Fig. 5). SND1 bound to the region encompassing −981 to −1536 bp upstream (*ABI4pro #1*) of the *ABI4* coding DNA sequence (CDS). We further characterized the SND1 binding site in the *ABI4* promoter region by dividing them into several small sections (Fig. 5A). We analyzed cis-elements of *ABI4* promoter region by PlantCARE program (http://bioinformatics.psb.ugent.be/webtools/plantcare/html/)^19^. By qRT-PCR, the −1386 to −1536 (q#1) and −1079 to −1179 (q#4) regions were identified to be important for SND1-*ABI4* promoter interaction (Fig. 5B). To confirm this result, we conducted a traditional yeast one-hybrid assay approach in which the *ABI4* promoter sequence (*ABI4pro #1*) was used as a bait. As shown in Fig. 5C, the SND1 protein exhibited successful binding to the *ABI4* promoter, whereas empty vector did not show any interaction. Moreover, the interaction between SND1 and *ABI4* promoter was confirmed via the electrophoretic mobility shift assay (EMSA). We generated the protein of the SND1 NAC domain containing 191 amino acids, and conducted EMSA with the probe of *ABI4* promoter. The *ABI4* promoter q#1, which includes ABA-responsive element (ABRE), was used for the EMSA. In this experiment, we also found that the SND1 NAC domain binds directly to the *ABI4* promoter, especially to the part of q#1 (−1386 to −1536 upstream from coding DNA sequence) (Fig. 5D). These results indicate that SND1 directly binds to *ABI4* promoter in *Arabidopsis*.

## Discussion

To actively cope with environmental threats, plants have developed various physiological and molecular mechanisms^20^. We have long been studying the responses of plants to environmental stress and have found that flavonoids play an important role in these resistance mechanisms^8^. In the present study, our findings reveal new functions of SND1 in ABA signalling. We hypothesized that reducing the lignin content via a knockout mutation of its master controller *SND1* might enhance abiotic stress tolerance. Contrary to our expectation, we observed that the *snd1ko* mutation lowered the anthocyanin content, whereas it increased in the *SND1* overexpression line (Fig. S1). As shown in Fig. S2, we observed altered expression of a variety of anthocyanin biosynthesis genes in both *snd1ko* and *SND1-OE* plants. These results suggest that SND1 plays a role in anthocyanin accumulation, besides it role in the synthesis of lignin^3^. However, the role of SND1 in anthocyanin biosynthesis has not been previously studied.

We investigated how the expression of *SND1* varies upon various treatments with hormones under different growing conditions. The results revealed that the most significant change in SND1 was caused by salinity stress (Fig. 1). This change in the expression of SND1 in response to salinity stress may correlate with the reduced survival rate of *snd1ko* plants in the presence of salt at high concentration in the medium (Fig. 2). One possibility is that the *snd1ko* plants accumulate less anthocyanins, which serve as antioxidants. Another possibility is that SND1 functions as a transcriptional regulator of other genes, such as some of the ABA signalling genes. To evaluate this hypothesis, we screened a number of genes by the qRT-PCR to identify genes with altered transcript level in *snd1ko*, in response to salinity (Fig. 3A). In this analysis, the *Myb46-OE*/*snd1ko* line was included, as we wanted to know if *Myb46*, one of the target genes of *SND1* in the lignin biosynthesis pathway^21^, can recover the altered phenotype of *snd1ko* when it is overexpressed in the *snd1ko* background. The results revealed that Myb46 is not involved in the SND1-driven alterations in terms of ABA related stress responses (Figs 3A and 4B). It has been reported that double knockout mutations of *MYB46* and *MYB83* result in secondary cell wall deficiency, thereby limiting plant growth^21^. Thus, the finding of the present study imply that SND1 likely drives different sets of genes to control ABA signalling and ABA biosynthesis.

In the search for SND1 target genes, we initially focused on the *NCED3* gene, based on the qRT-PCR results (Fig. 3B). The *NCED* genes encode 9-cis-epoxycarotenoid dioxygenase, which catalyzes the cleavage of 9-cis-epoxycarotenoid to xanthoxin in the key regulatory ABA biosynthesis step^22^. NCED3 is important for ABA biosynthesis during drought^23^, as evidenced by the fact that *nced3* mutants exhibited increased water loss and low ABA content^23^. Thus, the *snd1ko* plants may accumulate more ABA than WT, as the NCED3 transcript level was found to be higher in this mutant (Fig. 3B). In accordance with this result, we observed that the *snd1ko* plants accumulated more ABA than WT (Fig. 4A). The *SND1*-complementation line exhibited the recovery of *snd1ko* in terms of ABA content. Moreover, enhanced ABA content in the *snd1ko* plants seems to confer increased germination sensitivity to ABA (Fig. 4B). However, by the ChIP analysis, we found that SND1 does not bind to the promoter of *NCED3* (data not shown). Thus, we performed the ChIP analysis with other possible candidate genes, which revealed that the *ABI4* gene is an SND1 target. As shown in Fig. 3A, the *ABI4* transcript level was enhanced in the *snd1ko* mutant. Our detailed ChIP analysis and yeast one hybrid assay clearly demonstrated that SND1 directly bound to the *ABI4* promoter (Fig. 5). Moreover, the result of EMSA showed that SND1 interacts with promoter fragment of −1386 to −1536 from *ABI4* coding DNA sequence. ABI4 also has been shown to positively regulate seed dormancy through ABA biosynthesis by directly binding to the promoters of *CYP707A1* and *CYP707A*2, thereby, repressing their expression^24^. *Arabidopsis CYP707A* encodes ABA 8′-hydroxylase, which plays key roles in ABA catabolism^25^, as indicated by the observation that the enhanced expression level of *CYP707A1* and *CYP707A2* in the *abi4 ko* mutant reduced ABA content^24^. However, we did not observe any difference in the transcript levels of *CYP707A1* and *CYP707A2* between in WT and the *snd1ko* mutant (data not shown). In contrast, ABI4 is known to increase the expression of *CHO1* which is an activator of *NCED3*^26^. Hence, the increase in ABA content in the *snd1ko* mutant was not caused by the suppression of *CYP707A1*/*2*, but by the genes *CHO1* and *NCED3*.

**Figure 6:**
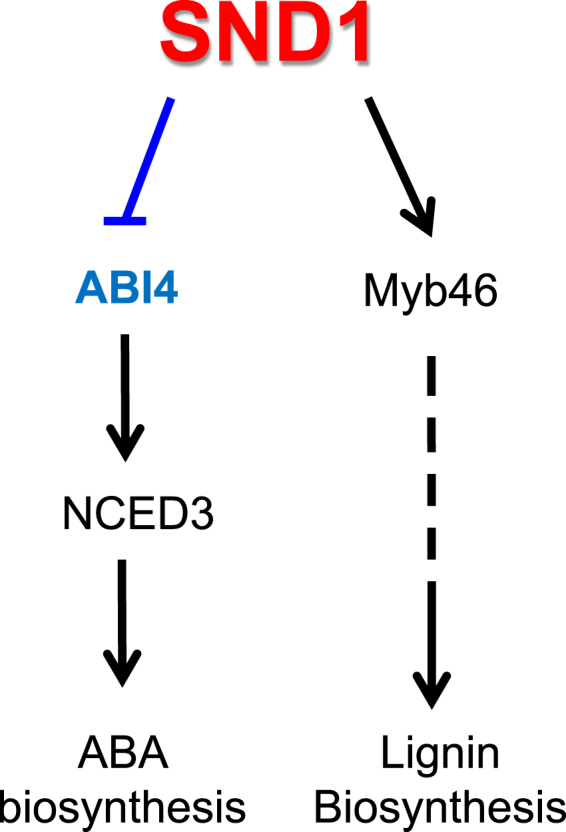
Working model of SND1, negatively regulating ABI4 in the ABA biosynthesis pathway. SND1 is a master switch for the formation of secondary cell walls through the induction of Myb46 under normal conditions. For a strong and successful plant growth, SND1 directly binds to the *ABI4* promoter and negatively regulates the expression of the *ABI4* transcript, inhibiting of the ABA biosynthesis pathway. SND1 is induced by osmotic stress and inhibits continuous ABA production under these conditions.

Abscisic acid frequently plays a central role in adaptation of plants to environmental stress^27,28^. In the present study, we demonstrated that SND1 has more than one function; it can reduce ABA biosynthesis via the repression of *ABI4* (Figs 3 and 5), and it can also enhance lignin biosynthesis through *Myb46* activation^21^. Furthermore, ABA has been shown to reprogram transcriptional schemes for improved adaptation to abiotic stress^29^. In this pathway, ABA bound to PYR/PYL/RCAR (PYRABACTIN RESISTANCE1/PYR1-LIKE/REGULATORY COMPONENTS OF ABA RECEPTORS) receptors, and inhibits ABI1, ABI2, HAB1, and PP2CA protein phosphatases, thus activating SnRK2 protein kinases^18^. In addition to triggering the stress defence pathways and closing of stomata, one important function of ABA is to inhibit seedling growth. A transcriptional mechanism associated with the inhibition of the cell cycle^30^ and metabolism^31^ has been proposed in association with ABA function. Moreover, ABA modulates the function of PM H^+^-ATPase^32^ and nutrient transporters to adjust of plant growth^33^. On the basis of our results, we propose a working model of the dual function of SND1 (Fig. 6). Under normal conditions, plants can allocate all available resources to the growing points; however, they have to redirect them under growth-limiting conditions, of one kind or another. Therefore, it is plausible to reason that SND1 activates Myb46, thereby fostering the biosynthesis of lignins to produce sufficient cell wall components needed for plant growth, while maintaining a low concentration of ABA, a growth inhibitor. Such dual function of SND1 may help plants to modulate their growth patterns to suit specific growth environmental conditions and, thus, thrive in the best possible way under such circumstances.

## Methods

### Plant material and growth conditions

The seed coat of all experimental materials was sterilized. After 3 d at 4 °C in dark, the seeds were sown on half-strength Murashige and Skoog (MS) medium supplemented with 2% sucrose (pH 5.7), as the normal growing condition. The seedlings were grown in a growth chamber at 23 °C and 60% relative humidity under long-day conditions (light 16 h, dark 8 h) for all experiments. *Arabidopsis thaliana* ecotype Colombia-0 was used as the wild type (WT). The *snd1* knockout mutant (SALK_015495.54.50.x) was obtained from the Arabidopsis Biological Resource Center (ABRC).

### DNA construction and generation of transgenic plants

Coding DNA sequences (CDS) of SND1 and Myb46 were amplified by the RT-PCR and cloned into the TOPO vector (pCR™8/GW/TOPO^®^ TA Cloning Kit, Invitrogen). The SND1-TOPO vector construct was subcloned into the pMDC32 vector^34^ to generate the SND1 overexpressing lines, and the pMDC83 vector^34^ for SND1-GFP fusion. The Myb46-TOPO vector construct was subcloned into the pMDC32 vector. For SND1 complementation, a DNA construct including the sequence −1000 bp upstream to the coding DNA sequence (CDS) was cloned into the TOPO vector. The vector was subcloned into the pMDC100 vector^34^ to generate the SND1 complementation line. The subcloning constructs were transformed into *Agrobacterium tumefaciens* (GV3101) by electroporation. The floral dipping method was used for plant transformation^35^. Background plant of the *SND1***-**overexpressing line and the *SND1 GFP* line was Col-0. The *Myb46-OE*/*snd1ko* and *SND1***-**complementation lines were generated in a *snd1* knockout mutant background.

### RNA isolation and quantitative real-time RT-PCR

The total RNA was isolated from eight-day-old seedlings following treatment with 10 µM ABA, 10 µM Indole-3-acetic acid (IAA), 10 µM Jasmonic acid (JA), 10 µM Salicylic acid (SA), 200 mM NaCl, 300 mM NaCl, 400 mM mannitol, or 200 mM sucrose for 6 h. The cDNA was synthesized using the total mRNA samples from eight-day-old seedlings and a cDNA synthesis kit (RevertAid First Strand cDNA Synthesis Kit, ThermoScientific). For quantitative real-time PCR (qRT-PCR), the cDNA was amplified using the EvaGreen MasterMix (BrightGreen qPCR MasterMix, Abm). Actin2 was used as an internal control; gene primers for the qRT-PCR are listed in Table S1.

### Salinity stress phenotype analysis

The seeds were sown on filter paper placed on half-strength MS medium supplemented with 2% sucrose (pH 5.7). Two days after seeding, the filter papers with seedlings were moved onto a medium supplemented with 200 mM NaCl.

### Determination of ABA content

The content of ABA was measured as previously described^36^. The samples of eight-day-old seedlings were used to extract ABA after treatment with 200 mM NaCl for 24 h. The content of ABA was measured using the Phytodetek Elisa kit according the instructions of the manufacturer.

### Analysis of cis-regulatory elements and chromatin immunoprecipitation

The cis-elements in *ABI4* promoter were analyzed by PlantCARE program (http://bioinformatics.psb.ugent.be/webtools/plantcare/html/)^19^. The chromatin immunoprecipitation (ChIP) was performed with modifications to the method originally reported^37^. Fourteen-day-old SND1GFP seedlings were used to extract nuclear content with cross-link. An antibody against GFP (G1544, Sigma, https://www.sigmaaldrich.com/) was used for immunoprecipitation. The purified DNA was quantified by the qRT-PCR using the specific primers listed in Table S1.

### Yeast one-hybrid assay

Full-length SND1 was fused in frame with the GAL4 activation domain of pGADT7 AD vector. The bait construct of ABI4 promoter region (ABI4pro Sac1 F: GAGCTCTAGTTTTTACTTATGTCCAAAAATATGA, and ABI4pro Kpn1 R: GGTACCTAGTAAAAGATCTAAATGCATTTTTAAT) was cloned into the pAbAi vector. The recombinant plasmid pGADT7-SND1 and ABI4 promoter bait plasmid were co-transformed into the yeast strain Y1HGold (Clontech) as described in the instruction of the manufacturer (Matchmaker^®^ Gold Yeast One-Hybrid Library Screening System, Clontech). The yeast containing pGADT7-empty vector and ABI4 promoter bait were used as control. The transformants were cultured in SD/-Leu medium, and then transferred to SD/-Leu medium supplemented with 50 ng/mL aureobasidin A (AbA).

### Protein purification and electrophoretic mobility shift assay

To obtain recombinant protein for the electrophoretic mobility shift assay (EMSA), the DNA fragment of SND1 NAC domain was subcloned into pMAL-His-C2X vector, which is a recombinant pMAL-C2X vector (NEW ENGLAND BioLabs). The 6x-histidine (His) tag was fused in-frame with the C-terminus of maltose-binding protein (MBP) of pMAL-His-C2X vector. The primer for SND1 NAC domain cloning is listed in Table S1. The construct obtained was transformed into *E-coli* strain BL21 (DE3). To express the recombinant protein, the recombinant *E-coli* was grown in LB liquid medium at 37 °C until the OD (600) reached 0.7, and then 0.1 mM isopropyl-b-D-thiogalactopyranoside was added to the medium and the cells were grown again at 18 °C for 18 h. After centrifugation at 3500 rpm for 1 h, the supernatant was removed and the bacteria were stored at −20 °C until the isolation of protein. The Ni-His tag column was used to purify the recombinant protein. The *AIB4* promoter fragment was amplified by the PCR with both 5′ and 3′ biotin-labelled primers. The primer sequences for the *ABI4* promoter fragment were similar to those of the *ABI4pro q#1* primer for the ChIP assay. The EMSA assay was conducted according to the instructions provided in the LightShift^®^ Chemiluminescent EMSA kit insert (Thermo Scientific).

### Germination rate analysis

The seeds were sown on half-strength MS medium supplemented with 2% sucrose (pH 5.7) or ABA-supplemented medium. Eight days after seeding, the roots were counted to analyse germination rates. One-hundred seeds were used for the analysis and three independent experiments were conducted.

### Statistical analyses

Each experiment was replicated at least thrice. The statistical analyses were performed by the one-way ANOVA, followed by Tukey’s test for comparison of means at 95% confidence level.

### Gene accession number

The accession numbers of the genes used in this study are as follows: ABF1 (AT1G49720), ABF2 (AT1G45249), ABF3 (AT4G34000), ABF4 (AT3G19290), ABI1 (AT4G26080), ABI2 (AT5G57050), ABI3 (AT3G24650), ABI4 (AT2G40220), ABI5 (AT2G36270), CesA8 (AT4G18780), COR15a (AT2G42540), CHI (AT3G55120), CHS (AT5G13930), DFR (AT5G42800), F3H (AT3G51240), F3′H (AT3G51240), FLS1 (AT5G08640), LDOX (AT4G22880), Myb46 (AT5G12870), NCED3 (AT3G14440), NDR1 (AT3G20600), NHL6 (AT1G65690), NST1 (AT2G46770), PAP1 (AT1G56650), RD29a (AT5G52310), RD29b (AT5G52300), SND1 (AT1G32770).

## Electronic supplementary material

Supplementary Information
