# Supplementary material for: Dual role of SND1 facilitates efficient communication between abiotic stress signalling and normal growth in Arabidopsis
Source: Sci Rep. 2018 Jul 4;8:10114. doi: 10.1038/s41598-018-28413-x (PMC6031621; doi:10.1038/s41598-018-28413-x)
Supplement: Supplementary file 1 — Supplementary Information [file 41598_2018_28413_MOESM1_ESM.docx]

**Dual role of SND1 facilitates efficient communication between abiotic stress signalling and normal growth in *Arabidopsis***

Chan Young Jeong^1, 2^, Won Je Lee^1^, Hai An Truong^1^, Cao Sơn Trịnh^1^, Joo Yeon Jin^1^, Sulhee Kim^1^, Kwang Yeon Hwang^1^, Chon-Sik Kang^3^, Joon-Kwan Moon^4^, Suk-Whan Hong^5,^ * and Hojoung Lee^1, 2,^ *

*^1^Department of Biosystems and Biotechnology, College of Life Sciences and Biotechnology, Korea University, Anam-dong 5-ga, Seongbuk-gu, Seoul 136-713, Republic of Korea.*

*^2^Institute of Life Science and Natural Resources, Korea University, Seoul 136-713, Republic of Korea.*

*^3^Crop Breeding Division, National Institute of Crop Science, RDA, 181 Hyeoksin-ro, Iseo-myeon, Wanju-gun, Jeollabuk-do, 54955, Korea.*

^4^*Department of Plant Life and Environmental Sciences, Hankyong National University, 327 Jungangro, Anseong, 17579, Republic of Korea.*

*^5^Department of Molecular Biotechnology, College of Agriculture and Life Sciences, Bioenergy Research Center, Chonnam National University, Gwangju, Republic of Korea.*

***Corresponding author:**

**Hojoung Lee**

Telephone: +82-2-3290-3006

Fax: +82-2-3290-3508

Email: [lhojoung@korea.ac.kr](mailto:lhojoung@korea.ac.kr)

**Suk-Whan Hong**

Telephone: +82-62-530-2180

Fax: +82-62-530-2180

Email: sukwhan@chonnam.ac.kr

**Supplementary methods**

**Plant material and growth conditions**

The seed coat of all experimental materials was sterilized. After 3 d at 4°C in dark, the seeds were sown on half-strength Murashige and Skoog (MS) medium supplemented with 2% sucrose (pH 5.7), as the normal growing condition. The seedlings were grown in a growth chamber at 23°C and 60% relative humidity under long-day conditions (light 16 h, dark 8 h) for all experiments. *Arabidopsis thaliana* ecotype Colombia-0 was used as the wild type (WT). The *snd1* knockout mutant (SALK_015495.54.50.x) and *nst1* knockout mutant (SALK_120377.53.15.x) were obtained from the Arabidopsis Biological Resource Center (ABRC).

**Anthocyanin measurement**

Four-day-old seedlings were treated with 0 or 200 mM sucrose for 24 h and used to extract anthocyanin contents. The samples were ground in liquid nitrogen and incubated with 300 µl methanol and 1% HCl at 4°C overnight in the dark. After adding 250 µl distilled water and 250 µl chloroform, the samples were centrifuged at 3000 rpm for 2 min. The supernatant was removed and placed into a new e-tube and the anthocyanin content was measured at OD 530 nm using a spectrophotometer.^1^

**DNA construction and generation of transgenic plants**

Coding DNA sequence (CDS) of SND1 was amplified by the RT-PCR and cloned into the TOPO vector (pCR™8/GW/TOPO^®^ TA Cloning Kit, Invitrogen). The SND1-TOPO vector construct was subcloned into the pMDC32 vector^2^ to generate the SND1 overexpressing lines. For SND1 complementation, a DNA construct including the sequence -1000 bp upstream to the CDS was cloned into the TOPO vector. The vector was subcloned into the pMDC100 vector^2^ to generate the SND1 complementation line. The subcloning constructs were transformed into *Agrobacterium tumefaciens* (GV3101) by electroporation. The floral dipping method was used for plant transformation^3^. Background plants of the *SND1*-overexpressing line were Col-0, whereas the *SND1*-complementation line was generated on a *snd1* knockout mutant background.

**Salinity stress phenotype analysis**

5 d after seeding on the normal medium, the seedlings were transferred onto medium supplemented with 200 mM NaCl. The survival rates were determined by counting the number of green cotyledons after 3, 4, and 5 d.

**RNA isolation and quantitative real-time RT-PCR**

The total RNA was isolated from eight-day-old seedlings following treatment with 200 mM sucrose or NaCl for 6 h. The cDNA was synthesized using the total mRNA samples from eight-day-old seedlings and a cDNA synthesis kit (RevertAid First Strand cDNA Synthesis Kit, ThermoScientific). For quantitative real-time PCR (qRT-PCR), the cDNA was amplified using the EvaGreen MasterMix (BrightGreen qPCR MasterMix, Abm). Actin2 was used as an internal control; gene primers for qRT-PCR are listed in Table S1.

**Determination of sodium content**

Two-week-old Col-0, *snd1ko* mutant, and *SND1*-overexpressing line seedlings were treated with 1/2 MS media containing 100 or 200 mM NaCl for 48 h and subsequently used to extract sodium. Following the addition of 2 mL distilled water to 50 mg of each sample, the sodium content was extracted. Each sample was filtered by ADVENTEC 5A filter paper and analysed at 1/100 dilution by PerkinElmer 8300 Series ICP.

**Statistical analyses**

Each experiment was replicated at least thrice. The statistical analyses were performed by the one-way ANOVA, followed by Tukey’s test for comparison of means at 95% confidence level.

**Supplementary Data**

**Table S1. Lists of primers for qRT-PCR.** These primers were used for qRT-PCR


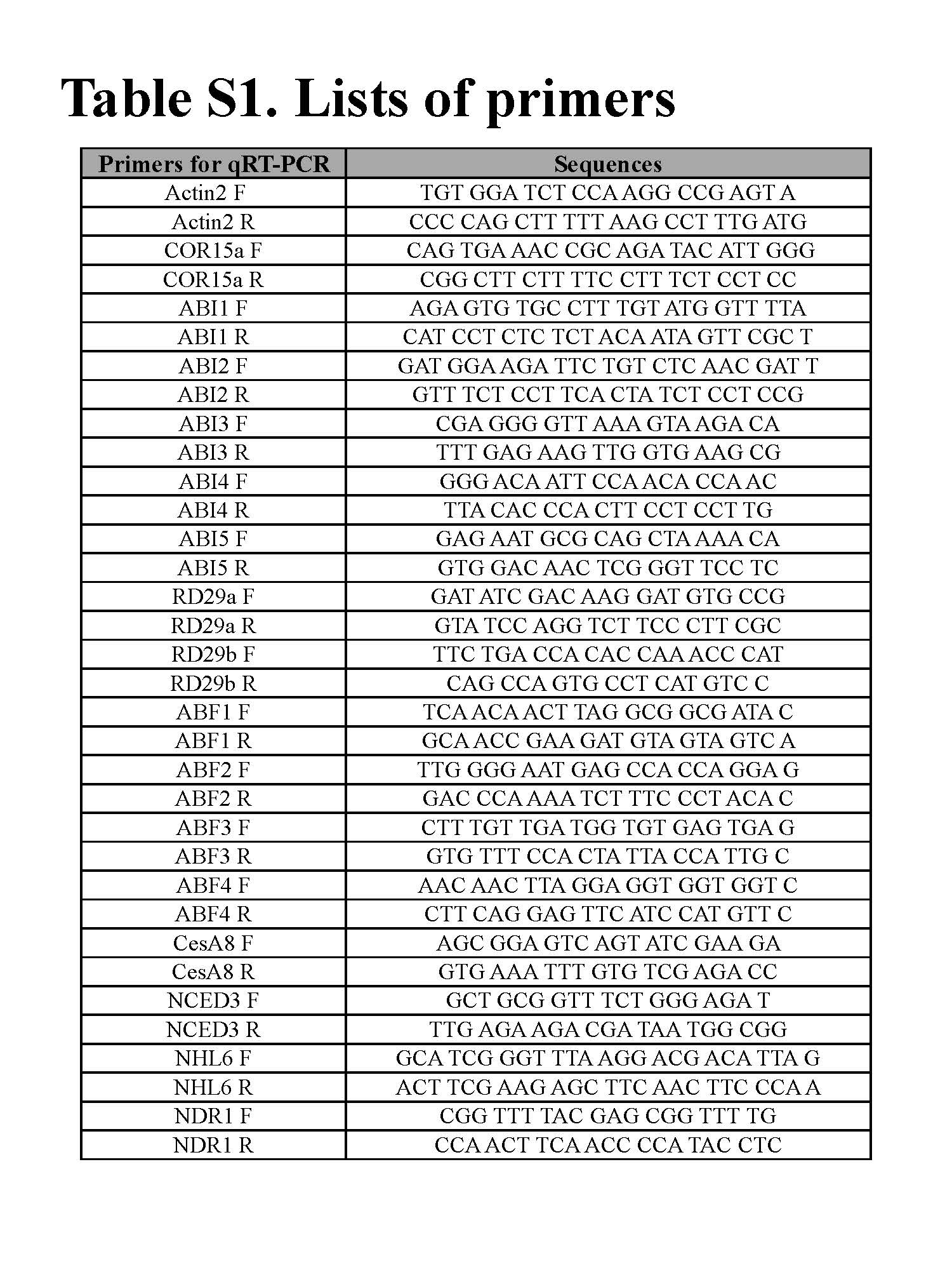


**Table S2. Lists of primers for qRT-PCR and ChIP.** These primers were used for qRT-PCR and Chromatin imunoprecipitation (ChIP) assay.


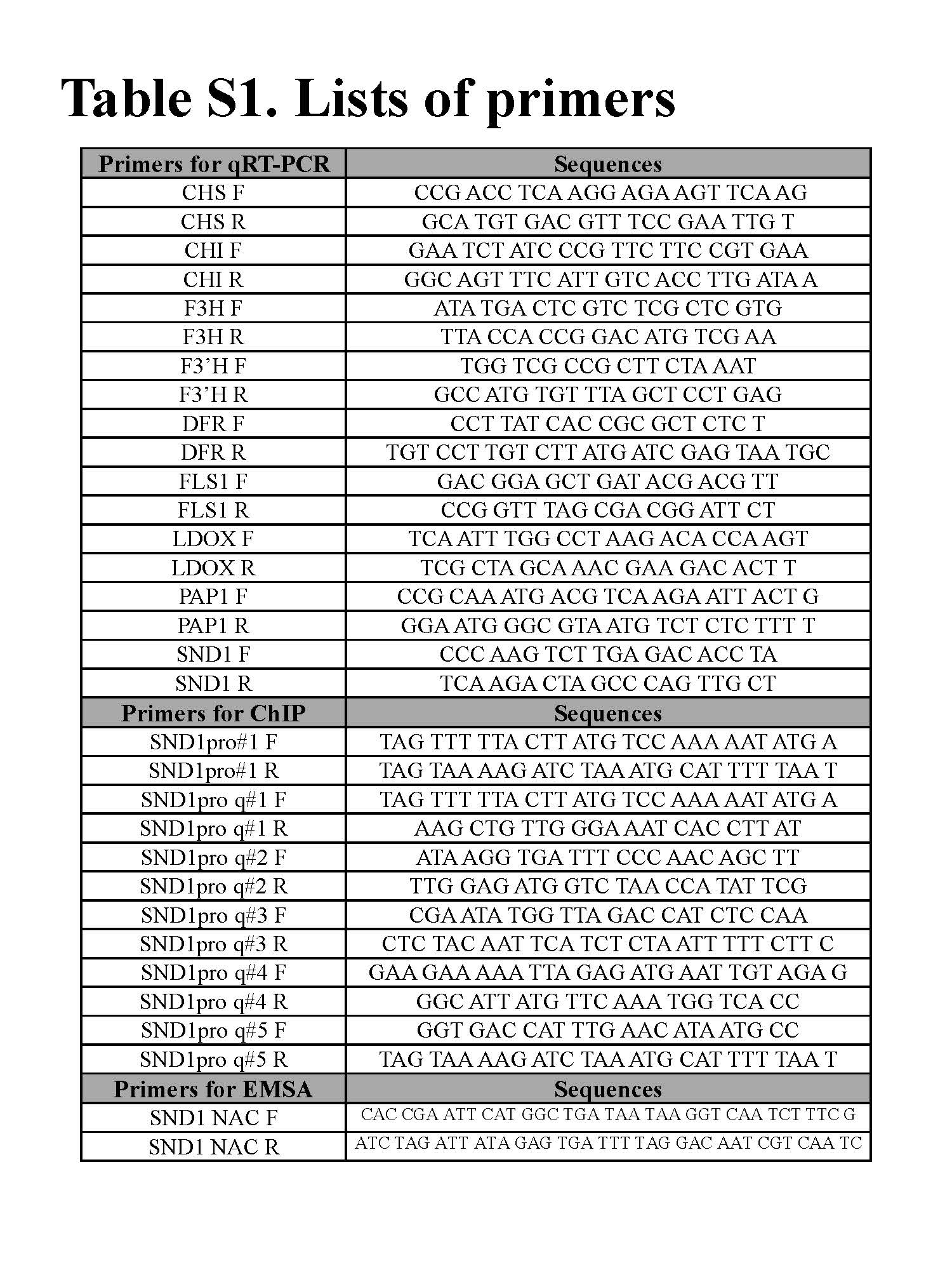


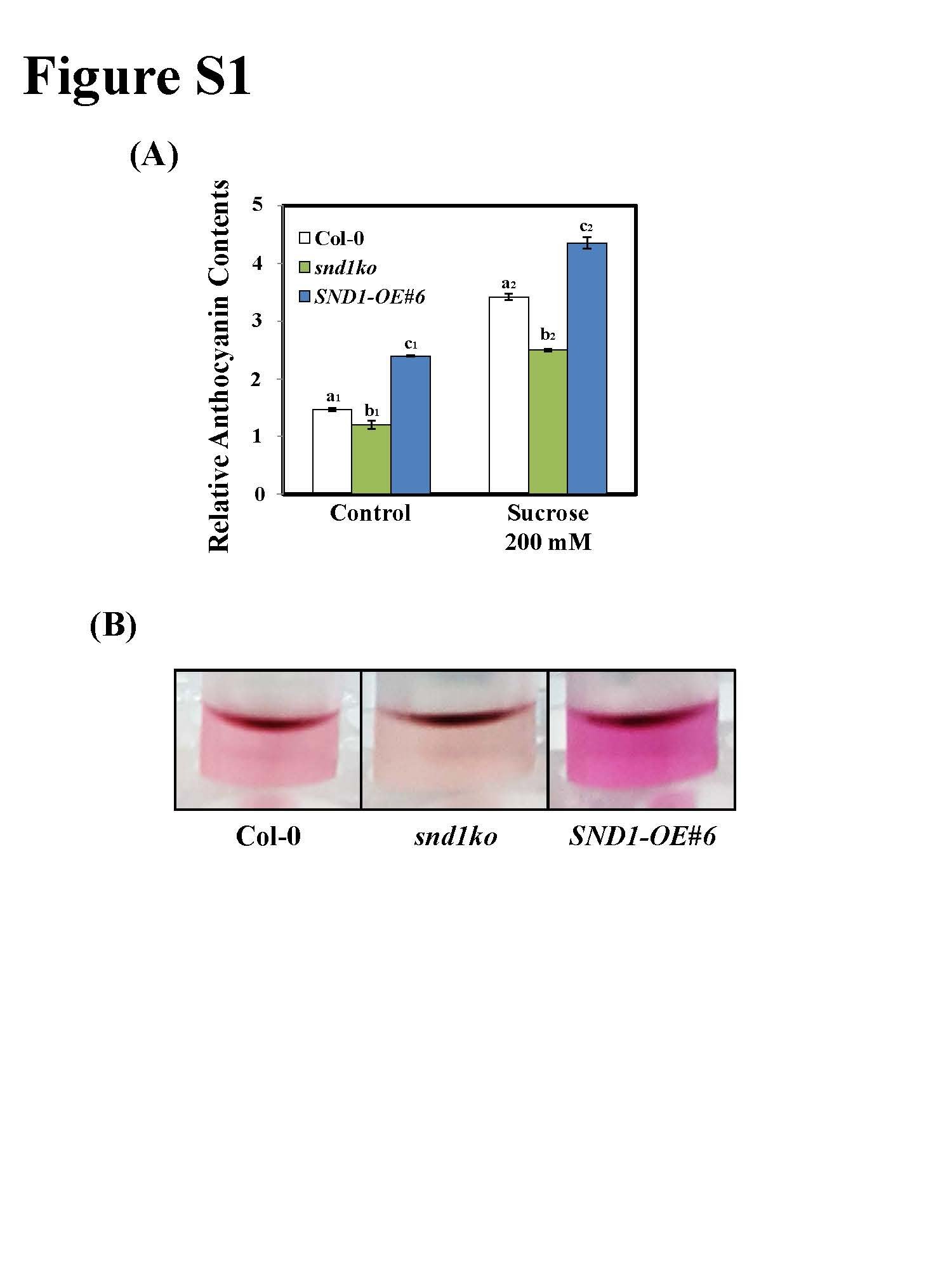


**Figure S1. Anthocyanin accumulation in Col-0, *snd1ko* mutant, and *SND1*-overexpressing line**

(A) The relative anthocyanin content of Col-0, *snd1ko* mutant, and *SND1*-overexpressing line. Four-day-old seedlings were used to extract anthocyanin following treatment with 200 mM sucrose for 24 h. The anthocyanin content was measured by a spectrophotometer at OD 530 nm. (B) The purple colour indicates anthocyanin accumulation in each sample. The error bars indicate the standard error (SE) of three replicates. The values with different letters were significantly different from that of WT plants (P < 0.05).


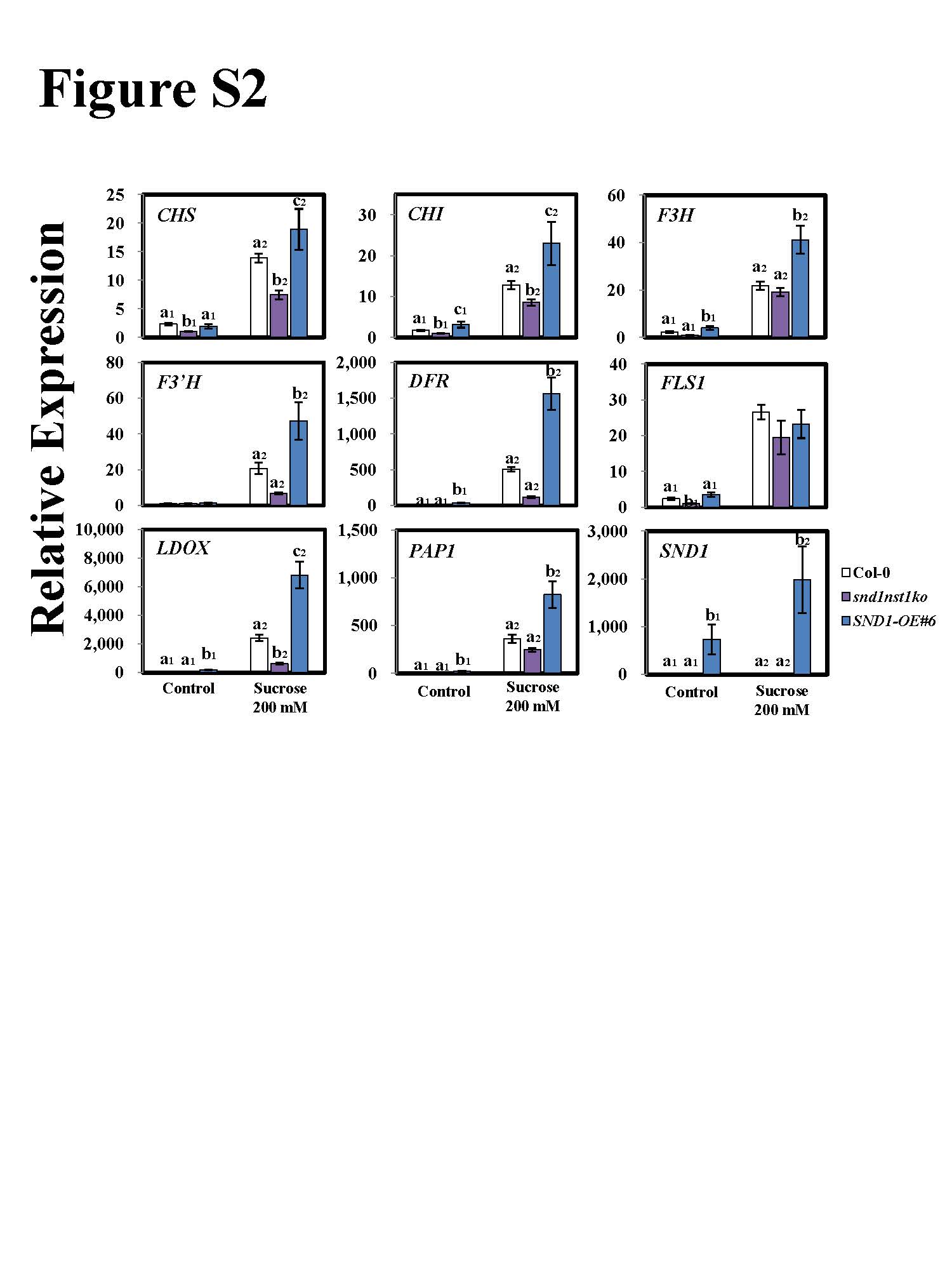


**Figure S2. Transcript level of genes related to flavonoid biosynthesis in Col-0, *snd1nst1ko* double mutant, and *SND1*-overexpressing line**

The relative expression of flavonoid-biosynthesis related genes in Col-0, *snd1nst1ko* double mutant, and *SND1*-overexpressing line were determined by the qRT-PCR. Eight-day-old seedlings were used to extract mRNA following treatment with 200 mM sucrose for 6 h. The error bars indicate the standard error (SE) of three replicates. The values with different letters were significantly different from that of WT plants (P < 0.05).


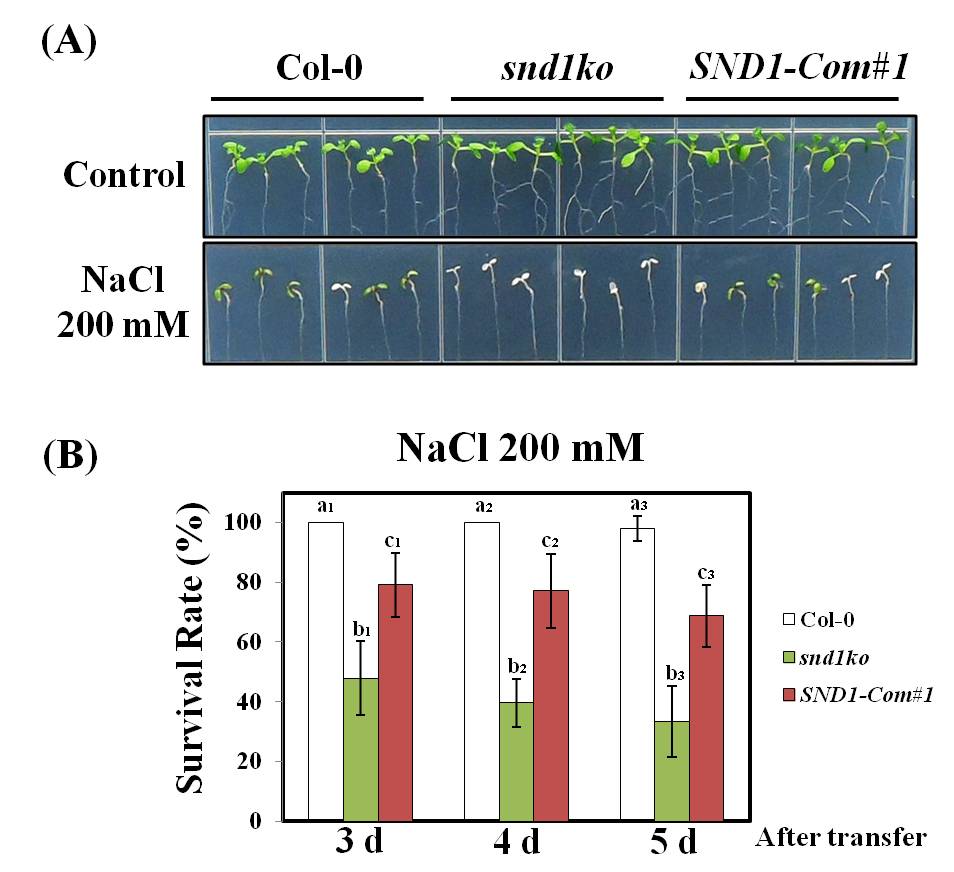


**Figure S3. Phenotype and survival rate of Col-0, *snd1ko* mutant, and *SND1*-complementation line under salinity stress**

(A) Five-day-old seedlings of Col-0, *snd1ko* mutant, and *SND1*-complementation line were transferred to medium supplemented with 200 mM NaCl. The figure shows seedlings 4 d after transfer. (B) The survival rate was quantified by counting the number of green cotyledons for each seedling. The experiments included 60 seedlings and the error bars indicate the standard error (SE) of three replicates. The values with different letters were significantly different from that of WT plants (P < 0.05).


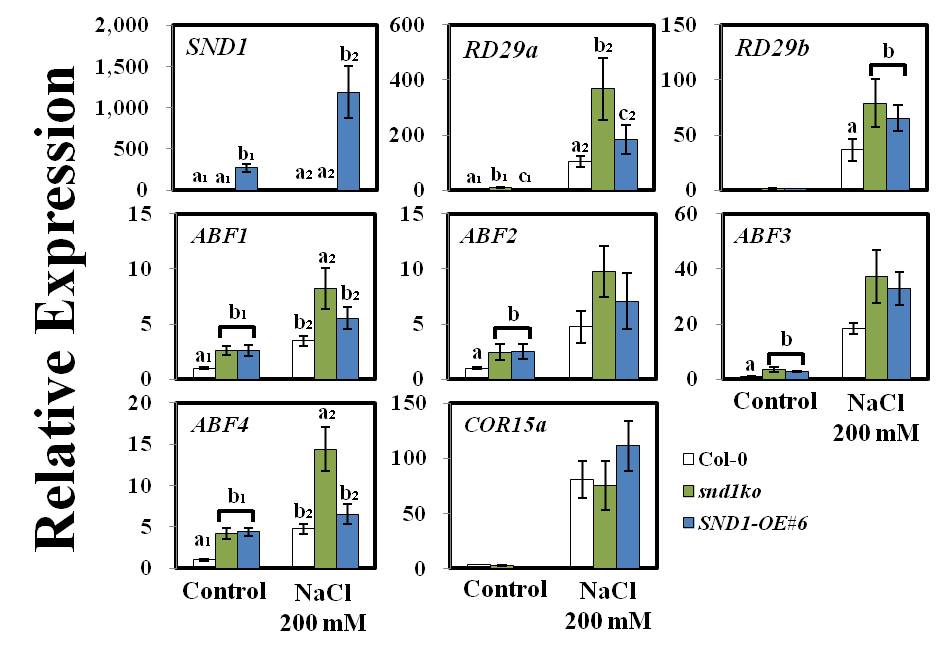


**Figure S4. Transcript level of ABA-related genes in Col-0, *snd1ko* mutant, and *SND1*-overexpressing line in response to salinity stress**

The relative expression of ABA-related genes in Col-0, *snd1ko* mutant, and *SND1*-overexpressing line was determined by the qRT-PCR. Eight-day-old seedlings were used to extract mRNA following treatment with 200 mM NaCl for 6 h. The error bars indicate the standard error (SE) of three replicates. The values with different letters were significantly different from that of WT plants (P < 0.05).


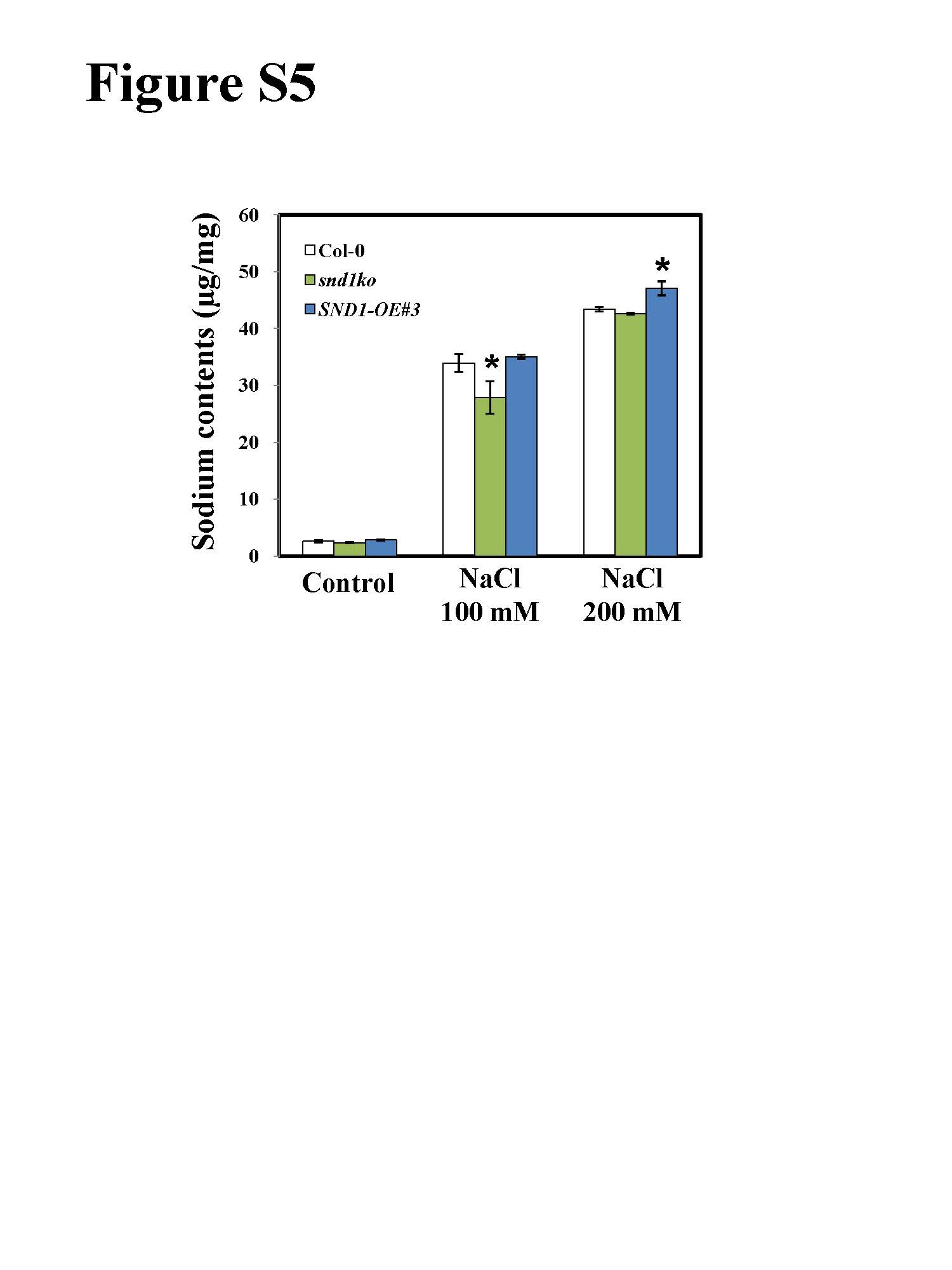


**Figure S5. Sodium Content in Col-0*, snd1ko* mutant, and *SND1*-overexpressing line under salinity stress**

Two-week-old seedlings were used to extract sodium content following treatment with 100 or 200 mM NaCl for 48 h. The error bars indicate the standard error (SE) of three replicates. The asterisks represent significant differences from that of Col-0 (P < 0.05).

**Reference**

1. Mancinelli, A.L., Hoff, A.M., & Cottrell, M. Anthocyanin production in Chl-rich and Chl-poor seedlings. *Plant Physiol* **86**, 652–654 (1998).

2. Curtis, M. D. & Grossniklaus, U. A gateway cloning vector set for high-throughput functional analysis of genes in planta. *Plant Physiol.* **133**, 462–469 (2003).

3. Clough, S. J. & Bent, A. F. Floral dip: a simplified method for *Agrobacterium*-mediated transformation of *Arabidopsis thaliana*. *Plant J.* **16**, 735–743 (1998).
